# Diet-related greenhouse gas emissions assessed by a food frequency questionnaire and validated using 7-day weighed food records

**DOI:** 10.1186/s12940-016-0110-7

**Published:** 2016-02-09

**Authors:** Camilla Sjörs, Sara E Raposo, Arvid Sjölander, Olle Bälter, Fredrik Hedenus, Katarina Bälter

**Affiliations:** Department of Medical Epidemiology and Biostatistics, Karolinska Institutet, SE-171 77 Stockholm, Sweden; Current address: Department of Nutrition, Harvard T.H. Chan School of Public Health, Boston, MA 02115 USA; KTH - Royal Institute of Technology, School of Computer Science and Communication, SE-100 44 Stockholm, Sweden; Stanford Graduate School of Education, Stanford, CA USA; Department of Energy and Environment, Chalmers University of Technology, SE-412 96 Gothenburg, Sweden; Stanford Prevention Research Center, Stanford School of Medicine, Stanford, USA

**Keywords:** Validation studies, Reproducibility of results, Food frequency questionnaire, Weighed food record, Epidemiology, Greenhouse gas emission, Climate change, Life cycle assessment, Carbon dioxide equivalents, Sustainable diets

## Abstract

**Background:**

The current food system generates about 25 % of total greenhouse gas emissions (GHGE), including deforestation, and thereby substantially contributes to the warming of the earth’s surface. To understand the association between food and nutrient intake and GHGE, we therefore need valid methods to assess diet-related GHGE in observational studies.

**Methods:**

Life cycle assessment (LCA) studies assess the environmental impact of different food items. We linked LCA data expressed as kg carbon dioxide equivalents (CO_2_e) per kg food product to data on food intake assessed by the food frequency questionnaire (FFQ) Meal-Q and validated it against a 7-day weighed food record (WFR). 166 male and female volunteers aged 20–63 years completed Meal-Q and the WFR, and their food intake was linked to LCA data.

**Results:**

The mean GHGE assessed with Meal-Q was 3.76 kg CO_2_e per day and person, whereas it was 5.04 kg CO_2_e using the WFR. The energy-adjusted and deattenuated Pearson and Spearman correlation coefficients were 0.68 and 0.70, respectively. Moreover, compared to the WFR, Meal-Q provided a good ranking ability, with 90 % of the participants classified into the same or adjacent quartile according to their daily average CO_2_e. The Bland-Altman plot showed an acceptable level of agreement between the two methods and the reproducibility of Meal-Q was high.

**Conclusions:**

This is the first study validating the assessment of diet-related GHGE by a questionnaire. The results suggest that Meal-Q is a useful tool for studying the link between food habits and CO_2_e in future epidemiological studies.

**Electronic supplementary material:**

The online version of this article (doi:10.1186/s12940-016-0110-7) contains supplementary material, which is available to authorized users.

## Background

The current production and distribution of food generates about 25% of total greenhouse gas emissions (GHGE), including deforestation, and thereby contributes to the warming of the earth’s surface [1]. There are technical mitigation options to reduce GHGE, but dietary shifts will be necessary if the climate target of a maximum 2° temperature increase is to be met with high certainty [2].

Although a healthy diet associated with low GHGE can meet nutritional requirements and may even bring health benefits compared to food habits with higher GHGE [3–7], not all low carbon diets are necessarily nutritious or leads to better health than a high carbon diet [8, 9]. Models of health impacts from various dietary scenarios, e.g. cardiovascular disease, cancer and overall mortality, suggest that healthy diets with lower GHGE are in line with general public health goals [10–12]. However, until now, most studies on diet, GHGE and health outcomes are based on theoretical models, and need to be confirmed in observational studies based on habitual food consumption.

In order to study climate-friendly diets, data on dietary intake need to be linked to data on GHGE from life cycle assessment (LCA) studies [13]. This raises the issue of valid dietary assessment methods for studying diet-related GHGE and health outcomes in large epidemiological studies. Lately, food frequency questionnaires (FFQs) have been used more frequently to estimate dietary GHGE. However, to our knowledge, this is the first study to specifically validate a FFQ’s ability to estimate dietary GHGE.

The aim of this study is to validate the assessment of GHGE from diet using a FFQ called Meal-Q compared to a 7-day weighed food record (WFR), as well as to evaluate the reproducibility of Meal-Q.

## Methods

The validation study VALMA (Validation of Methods Assessing diet and physical activity) has been described in detail elsewhere [14]. In brief, recruitment to the VALMA study took place in April 2009 in Stockholm County, Sweden, through public advertisements to the general population and students at universities. To be included, participants needed to have access to the Internet and an email address, not be trying to lose weight, not be pregnant and not have given birth within the past ten months. In total, 180 healthy male and female volunteers aged 20–63 years were enrolled. All participants provided informed consent. The questionnaires were sent out via e-mail and individual user names and passwords served as identifiers.

During the study, all participants responded to Meal-Q once and completed a 7-day WFR. About half of the participants were asked to fill out Meal-Q a second time, three weeks after the first, to enable evaluation of reproducibility.

Exclusions were made due to dropout (*n* = 1), illness (*n* = 2) and energy underreporting (*n* = 11), leaving 166 participants for validation analyses, and 87 participants for analyses of reproducibility. There were no statistically significant differences in age, body mass index (BMI, kg/m^2^), education level, fulltime workers, students, nutrition background, smokers and Swedish snuff users between included and excluded participants (data not shown).

The study was approved by the Research Ethics Committee at Karolinska Institutet.

Previous validation studies based on Meal-Q show that the questionnaire performs well regarding energy, macronutrients [14], fiber and micronutrients [15], although there were substantial variations for different nutrients. Crude daily intakes were overall higher in the 7-day WFR compared to Meal-Q. The energy-adjusted and deattenuated correlation coefficients ranged from 0.16 to 0.73, (e.g. 0.18 for energy and 0.33, 0.65 and 0.57 for protein, carbohydrates and total fat, respectively). The proportion of participants classified into the same or adjacent quartile ranged from 69 to 90%, (e.g. 70 % for energy and 76, 82 and 78% for protein, carbohydrates and total fat, respectively).

### Food frequency questionnaire (FFQ)

Meal-Q is a web-based FFQ assessing habitual dietary intake during the past few months, which includes pre-defined food items and intake frequencies. Respondents only fill in the food items that they eat at least once a month. The interactive format includes 102–174 food items, depending on the number of follow-up questions. To assess portion sizes of cooked dishes and vegetables the respondents choose between five photos with different amounts for each of the three following food groups: rice/potatoes/pasta, meat/chicken/fish/vegetarian substitutes, and raw/cooked vegetables. Standard portion sizes were used for all other food items.

### 7-day weighed food records (WFR)

At an introductory meeting, the participants received a household scale and oral and written instructions on how to record everything they ate and drank. During the WFR, participants reported their daily food intake through a web-based program covering more than 2000 food items. All WFRs were checked for completeness by the study personnel. Participants also recorded their physical activity by reporting the number of daily steps from a pedometer, as well as activities not captured by pedometers. This information was used to calculate each participant’s physical activity level (PAL) to identify potential under-reporters of energy intake in the WFR.

### Diet-related GHGE

We collected data from LCA studies for 65 food products or groups aiming at representing the average consumption pattern in Sweden (for LCA data see Additional file 1: Table S1).

The main diet-related greenhouse gases are methane, nitrous oxide and carbon dioxide. Methane is emitted from ruminants, rice cultivation and manure management, nitrous oxide from agriculture and manure management and carbon dioxide from transports and energy use during food production and processing.

All LCA studies included GHGE from agriculture and its inputs, and the majority also included emissions up to and including the retail phase. We adjusted all LCA data to include the same system boundaries, for example added standard emission factors from post-farm processes, including processing, packaging, distribution and retail [16]. Emissions after the retail phase (from transports, storing and cooking, as well as from waste management) were not included.

Carbon dioxide, methane and nitrous oxide have different global warming potential (GWP), and their combined effect is often presented as kg carbon dioxide equivalents (CO_2_e) per kg of food product [13]. The GWP used to calculate the CO_2_e was 1 for carbon dioxide, 34 for methane and 296 for nitrous oxide [17].

Portion sizes were reported in the form in which the products were consumed in both Meal-Q and WFR. We therefore recalculated LCA data for appropriate food items to the prepared form, considering both hydration, i.e. cooking of rice, and dehydration, i.e. cooking of meat [18].

We adjusted for unavoidable food losses (i.e. shell and bone) using data from the Swedish food composition database [18]. In addition we adjusted for avoidable food losses both before and after food preparation using data from the British Waste and Resources Action Programme [19] and a FAO report [20].

To calculate the CO_2_e for mixed dishes, up to three main food products or groups were weighted using standard recipes. Recipes from the Swedish food composition database were used when available [18]. Thereafter, LCA data on CO_2_e per kg food item were linked to all food items in Meal-Q and the WFR.

### Statistical analyses

Descriptive results are presented as means and standard deviations (SD) or numbers of participants (n) and percentages (%). The Wilcoxon-Mann–Whitney test was used to assess potential differences in age and BMI between included and excluded participants as well as between women and men. Further, Fisher’s exact test was used to assess potential differences regarding education level, fulltime workers, students, nutrition background, smokers and Swedish snuff users.

The Goldberg cut-off method [21] was used to identify energy under-reporters. The cut-off value was calculated using the energy intake from the WFR together with the obtained PAL values from the physical activity record.

The residual method was used to adjust CO_2_e for total energy intake and a constant (the CO_2_e at the mean energy intake from Meal-Q) was added to the residuals [22]. Means, medians and interquartile ranges (IQRs) of crude and energy-adjusted CO_2_e were compared between Meal-Q and the WFR. To test the ranking agreement and magnitude of misclassification of Meal-Q compared to the WFR, quartile cross-classifications were used. Participants were divided into quartile categories of crude and energy-adjusted CO_2_e and the proportion of participants classified into the same, adjacent, and extreme quartiles were calculated. To evaluate absolute agreement and potential differences in bias within the CO_2_e range, the method of Bland and Altman was used, where the differences in CO_2_e between Meal-Q and WFR were plotted against the average of the two methods [23]. The plot provides a measure of variation represented by the limits of agreement, i.e., ±2 SD of the mean difference. Besides the Bland-Altman plot, a scatter plot was also used as graphical evaluation of the associations.

Spearman and Pearson correlation coefficients were used to measure the degree of association between Meal-Q and the WFR. For Pearson correlation coefficients the data were log transformed to improve the normality of the distribution. To remove effects of within-person variation in the WFR, Pearson and Spearman correlation coefficients were deattenuated, using the formula of Beaton et al. [24] and Liu et al. [25]. The method of Willett and Rosner [26] was used to produce confidence intervals. The Wilcoxon signed rank test was used to test for differences between the methods.

Crude and energy-adjusted CO_2_e were compared between the first and second Meal-Q using the mean, median and IQRs. To evaluate the reproducibility, crude and energy-adjusted quartile cross-classifications of the first and second Meal-Q were made, as well as a scatter plot and a Bland-Altman plot. The Wilcoxon signed rank test was used to test for differences between the methods [23]. In addition, one-way ANOVA with random effects was used to compute intraclass correlation coefficients (ICCs) [27].

The significance level was set to α = .05. All *p*-values were two-sided. Analyses were performed using STATA 13.1 (STATA Corporation, College Station, TX, USA).

## Results

The characteristics of the participants are shown in Table 1. The mean age was 33 years, a majority of the participants had more than 12 years of education and almost 60 % were students. Women had a lower BMI than men (22.9 vs 23.8) and more men than women used Swedish snuff. There were no statistically significant differences between gender with regards to age, education, working full time, students, nutrition background or smoking.

**Table 1:** Characteristics of participants included in the validation analysis (*n* = 166)

Characteristics	All	Women	Men	
	(*n* = 166)	(*n* = 132)	(*n* = 34)	
	Mean (SD)	Mean (SD)	Mean (SD)	*P*-value
Age (years)	32.9 (11.6)	32.9 (11.9)	32.6 (10.3)	0.9106
BMI (kg/m^2^)	23.1 (3.6)	22.9 (3.8)	23.8 (2.2)	0.0068
	n (%)	n (%)	n (%)	*P*-value
Education > 12 years	133 (80)	106 (80)	27 (79)	1.000
Working full time	55 (33)	43 (33)	12 (35)	0.839
Student	97 (58)	79 (60)	18 (53)	0.559
Nutrition background^a^	49 (30)	43 (33)	6 (18)	0.097
Smoking^b^	11 (7)	6 (5)	5 (15)	0.098
Swedish snuff use^bc^	11 (7)	4 (3)	7 (21)	0.000

*BMI* Body mass index

^a^Working or studying in the field of nutrition. ^b^Data missing for three women. ^c^Swedish snuff (snus) is a moist powder tobacco product

The GHGE assessed by the WFR was 5.04 kg CO_2_e and statistically significantly higher than the 3.76 kg CO_2_e assessed by Meal-Q (Table 2). When participants were divided into quartiles according to their crude and energy-adjusted daily average CO_2_e, 42.8 and 47.6 % of the participants were classified into the same quartile. The proportions classified into the same or adjacent quartile were 81.3 and 90.4 % respectively for crude and energy-adjusted daily average CO_2_e, while 3.6 and 0.6 % were classified into extreme quartiles.

**Table 2:** CO_2_e for participants included in the validation analysis

	Crude kg CO_2_e per day			Energy-adjusted kg CO_2_e per day		
	Mean	Median	(IQR)	Mean	Median	(IQR)
WFR	5.04	4.47	(2.67)	5.04	4.81	(2.36)
Meal-Q	3.76	3.51	(1.83)	3.76	3.55	(1.69)
% of WFR^a^	75	78		75	74	

Mean, median and IQR of daily CO_2_e for participants included in the validation analysis (*n* = 166)

*IQR* Interquartile range, *CO*_*2*_ *e* Carbon dioxide equivalents, *WFR* Weighed food record. ^a^Meal-Q /WFR. There was a statistically significant difference in CO_2_e between WFR and Meal-Q. Crude *P* = 0.00, Energy-adjusted *P* = 0.00, for Wilcoxon signed rank test

**Fig. 1:**
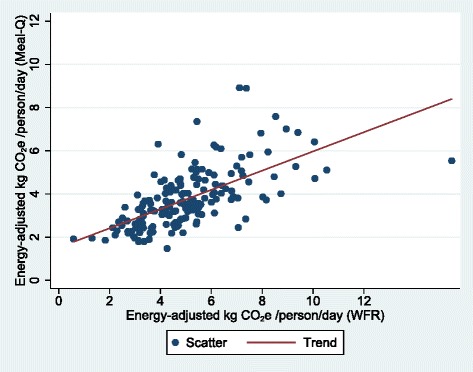
Scatter plot with energy-adjusted CO_2_e assessed by Meal-Q on the vertical axis and energy-adjusted CO_2_e assessed by WFR on the horizontal axis, for participants included in the validation analysis (*n* = 166). The outlier to the right is a person on a low carbohydrate high fat diet. The scatter plot for crude CO_2_e was similar (see Additional file 1: Fig. S1). CO_2_e, carbon dioxide equivalents. WFR, weighed food record

**Fig. 2:**
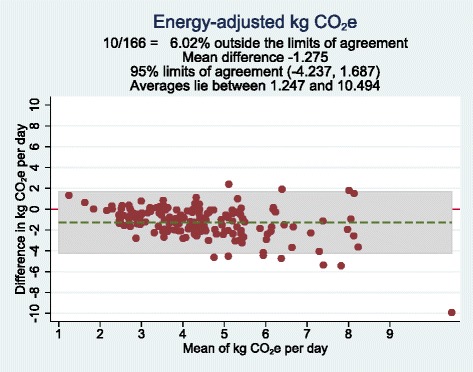
Bland-Altman plot showing the difference in energy-adjusted CO_2_e assessed by Meal-Q and the WFR plotted against the mean of the two methods, for participants included in the validation analysis (*n* = 166). Each data point represents one subject. The grey background show the 95% limits of agreement. The Bland-Altman plot for crude CO_2_e was similar (see Additional file 1: Fig. S2). CO_2_e, carbon dioxide equivalents. WFR, weighed food record

A scatter and a Bland-Altman plot of energy-adjusted daily CO_2_e assessed with Meal-Q and WFR are displayed in Figs. 1 and 2, respectively. The plots visualize to what extent there are differences in assessment of CO_2_e, comparing Meal-Q to the WFR. The scatter plot showed somewhat lower CO_2_e by Meal-Q compared to the WFR. The Bland-Altman plot showed that Meal-Q had a daily mean underestimation of about −1.28 kg CO_2_e and a trend of increasing underestimation with increasing CO_2_e. The scatter and Bland-Altman plots for crude daily CO_2_e were similar (see Additional file 1: Fig. S1 and S2).

Table 3 shows Pearson and Spearman correlation coefficients. The crude, energy-adjusted, and deattenuated Pearson correlation coefficients were 0.56, 0.67 and 0.68, respectively and the Spearman correlation coefficients were 0.56, 0.69 and 0.70 respectively.

**Table 3:** Pearson, Spearman and intraclass correlation coefficients of daily CO_2_e

	Crude (95 % CI)	Energy-adjusted (95 % CI)	Energy-adjusted and deattenuated (95 % CI)
Pearson corr. coefficients^a,b^	0.56 (0.46, 0.66)	0.67 (0.59, 0.76)	0.68 (0.59, 0.76)
Spearman corr. coefficients^b^	0.56 (0.44, 0.67)	0.69 (0.61, 0.77)	0.70 (0.61, 0.77)
Intraclass corr. coefficients^c^	0.72 (0.60, 0.81)	0.81 (0.73, 0.87)	

Pearson correlation coefficients and Spearman correlation coefficients of daily CO_2_e between Meal-Q and the WFR. Intraclass correlation coefficients of daily CO_2_e between first and second Meal-Q

*CO*_*2*_ *e* Carbon dioxide equivalents, *WFR* Weighed food record

^a^Log transformed data. ^b^Validity analyses, *n* = 166. ^c^Reproducibility analyses, *n* = 87

The crude daily mean CO_2_e assessed with the first Meal-Q was 3.86 kg CO_2_e compared to 3.87 kg CO_2_e with the second Meal-Q for the 87 participants included in the reproducibility analysis (Table 4). The differences in crude and energy-adjusted emissions between the first and second Meal-Q were not statistically significant. When participants were divided into quartiles according to their crude and energy-adjusted daily average CO_2_e with the first and second Meal-Q, 56.3 % and 63.2 % of the participants were classified into the same quartile. The proportions classified into the same or adjacent quartile were 88.5 % and 94.3 % respectively for crude and energy-adjusted daily CO_2_e, while 2.3 % and 1.1 % were classified into extreme quartiles. Crude and energy-adjusted intraclass correlation coefficients (ICCs) were 0.72 and 0.81 respectively (Table 3).

**Table 4:** CO_2_e assessed by first and second Meal-Q for participants in the reproducibility analysis

	Crude kg CO_2_e per day			Energy-adjusted kg CO_2_e per day		
	Mean	Median	(IQR)	Mean	Median	(IQR)
First Meal-Q	3.86	3.55	(2.20)	3.86	3.58	(1.87)
Second Meal-Q	3.87	3.64	(2.06)	3.87	3.37	(1.94)
Difference^a^	0.01	0.01		0.01	0.04	

Crude and energy-adjusted daily mean, median and IQR of daily CO_2_e assessed by first and second Meal-Q for participants in the reproducibility analysis (*n* = 87)

*IQR* Interquartile range, *CO*_*2*_ *e* Carbon dioxide equivalents

^a^Individual differences between the first and second Meal-Q. There was no statistically significant difference in CO_2_e between first and second Meal-Q. Crude *P* = 0.58, energy-adjusted *P* = 0.49, for Wilcoxon signed rank test

**Fig. 3:**
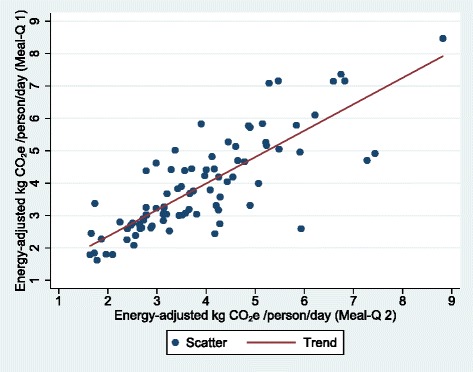
Scatter plot with energy-adjusted CO_2_e assessed by the first Meal-Q on the vertical axis and energy-adjusted CO_2_e assessed by the second Meal-Q on the horizontal axis, for participants included in the reproducibility analysis (*n* = 87). The scatter plot for crude CO_2_e was similar (see Additional file 1: Fig. S3). CO_2_e, carbon dioxide equivalents

**Fig. 4:**
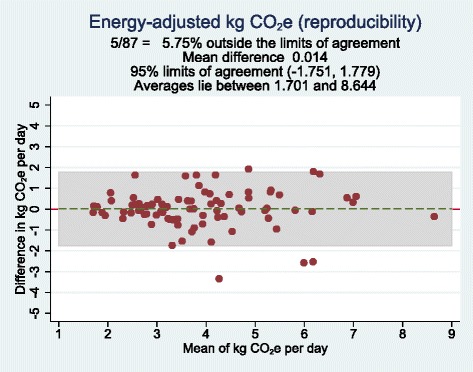
Bland-Altman plot showing the difference in energy-adjusted CO_2_e assessed by the first and second Meal-Q plotted against the mean of the two methods, for participants included in the reproducibility analysis (*n* = 87). Each data point represents one subject. The grey background show the 95 % limits of agreement. The Bland-Altman plot for crude CO_2_e was similar (see Additional file 1: Fig. S4). CO_2_e, carbon dioxide equivalents

A scatter and a Bland-Altman plot of energy-adjusted daily CO_2_e from the first and second Meal-Q are displayed in Figs. 3 and 4, respectively. The scatter plot showed slightly higher CO_2_e at higher emissions for the second Meal-Q compared to the first. The Bland-Altman plot showed a near zero mean difference between the questionnaires and equal estimations over the CO_2_e range. The scatter and Bland-Altman plots for crude daily CO_2_e were similar (see Additional file 1: Fig. S3 and S4).

## Discussion

Foods associated with high GHGE are mainly meat and dairy products [2], but there are large variations within these food groups. For example, the CO_2_e for one kg of beef is almost 48 kg CO_2_e after taking unavoidable and avoidable waste and food preparation into account. The corresponding value for one kg of poultry is about 4 kg CO_2_e. Despite the fact that Meal-Q is a relatively short questionnaire, we managed to capture the varying contribution of CO_2_e from different sources, which is shown in the validation analyses. Also, the good ability to rank individuals according to CO_2_e makes the method suitable for future epidemiological association studies, where accurate ranking is more important than the absolute magnitude of exposure [22]. Furthermore, results from the repeated assessments of Meal-Q show that the method has high reproducibility.

This is the first study validating diet-related GHGE from a FFQ compared to a 7-day WFR, therefore, comparisons with previous studies are difficult. However, there are studies evaluating the assessment of specific food products, such as beef and cheese. Overall, our results regarding diet-related GHGE are in line with results from validation studies on food products that are associated with high GHGE with regards to correlation coefficients, Bland-Altman plots, classification into the same or adjacent quintile and reproducibility [28–32].

To validate a new dietary assessment method is a challenge since the methods used differ from each other in several ways and perfect agreement between the two methods cannot be expected. Simultaneously, it is these inherent differences that make them suitable for validation studies, due to their largely independent errors. Meal-Q is a short user-friendly web-questionnaire that assesses habitual dietary intake retrospectively during the past few months, and only takes, on average, about 17 min to complete [14]. It takes advantage of an interactive design with extensive skip patterns, pre-defined food items and intake frequencies, uses standard portion sizes for most items, and only offers limited possibilities to personalize portion size for cooked dishes using photos. WFR on the other hand, aims to assess the total consumption of food and beverages during seven specific days. The dietary assessment is prospective, open-ended and personalized, but the participation burden is high, as the participants need to weigh and record everything they consume for a week. Given how different the two methods are, one would expect the estimates of the absolute diet-related GHGE to differ. This is also confirmed in the plots, where the energy-adjusted Bland-Altman plot shows that Meal-Q underestimates CO_2_e compared to WFR. Also, the underestimation increased as CO_2_e increased. There could be several reasons to why Meal-Q underestimates higher emissions, such as limited number of food items, that the largest intake frequencies were not high enough, that the portion sizes shown in the photos were too small or that the standard portion sizes used for the rest of the food items were too small to accurately assess the intake for some people. However, the data from the FFQ in epidemiological studies is used to rank individuals according to their exposure to enable risk comparisons between exposure groups, and the ranking of individuals according to their CO_2_e showed good agreement in the present study. In future epidemiological studies, Meal-Q will be used to assess diet-related CO_2_e and rank individuals with regard to their CO_2_e. The prevalence of various health outcomes will thereafter be compared between groups with food habits that contribute to high and low levels of CO_2_e, respectively, in order to study if climate-friendly food habits are also healthy food habits.

The study benefits from having LCA data for a large number of food items. Although there are inevitable uncertainties with LCA data, we have similar system boundaries for all food items. Also, as suggested by a recent review [33], we corrected the LCA values for weight change during food preparation, for example making the CO_2_e for rice correspond to cooked instead of dry rice, and compensated for unavoidable as well as avoidable food losses both before and after food preparation. To separate the contribution of CO_2_e from different meats, Meal-Q included questions on how often the participants consume the following meat products: chicken, beef, pork, ground meat dishes, bacon, lamb and game, offal, and hamburgers, respectively. Moreover, for ground meat dishes, such as meatballs, we used a mix of beef and pork based on standard recipes from the Swedish food composition database. Dairy products included in the questionnaire were milk, yoghurt, hot cocoa, cheese (hard and soft, respectively), ice cream, and dishes rich in dairy products such as pancakes and pizza. One uncertainty lies in the handling of mixed dishes, such as lasagna. All mixed dishes were divided into up to three main food products or groups and the CO_2_e values were weighted based on these. We have not evaluated the sensitivity of this approach and it is a potential source of bias. However, we estimated that the three main food products or groups in mixed dishes would be sufficient to assign an average LCA value.

Strengths in the study include its large sample size, only a few dropouts and high compliance with all parts of the study. Acknowledging that the participants were self-selected, highly educated, mainly female and that several had a background in nutrition, they were likely more motivated to be precise and complete the study than a random sample of the population would have been. Even though this contributes positively to the internal validity, it may decrease the external validity, i.e. the ability to generalize to the whole Swedish population. The short time period between the first and second Meal-Q makes changes in participant’s dietary habits less likely. However, if participants remembered their earlier answers, then reproducibility may have been overestimated. Also, true changes in dietary intake cannot be separated from measurement errors in reproducibility analyses [22]. While a low reproducibility would be a clear sign that the questionnaire is unsuitable to measure long-term intake, a high reproducibility does not prove correctness of the questionnaire, seeing that it may be as a result of correlated errors in the first and second administration of the questionnaire. Moreover, both Meal-Q and the WFR were web-based which enhance the quality of the data due to reduction of coding errors and missing data. Also, assessment of physical activity made us able to identify and exclude under-reporters of energy.

Here we present two types of correlation coefficients, although, their use in validation studies is disputed due to the risk of being misleading since they measure the linear relationship between two methods rather than the absolute agreement between them [22]. Therefore, to give a more nuanced picture, we also included Bland-Altman plots and scatter plots, along with cross-classification analyses.

Recent studies estimated diet-related GHGE using FFQs in the European Prospective Investigation into Cancer and Nutrition (EPIC) cohort [34, 35] and the Adventist Health Study 2 [36]. This is an emerging line of research and highlights the need for dietary assessment methods validated specifically with regards to CO_2_e.

## Conclusions

This is the first study validating the assessment of diet-related GHGE by a short FFQ. The results suggest that Meal-Q is a useful tool for studying the link between diet and CO_2_e in future epidemiological studies.

## Additional file

Additional file 1: Table S1. Data from life cycle assessment (LCA) studies expressed as carbon dioxide equivalents (CO_2_e) per kg of food product. **Fig. S1**. Scatter plot with crude CO_2_e assessed by Meal-Q on the vertical axis and crude CO_2_e assessed by WFR on the horizontal axis, for participants included in the validation analysis. **Fig. S2**. Bland-Altman plot showing the difference in crude CO_2_e assessed by Meal-Q and the WFR plotted against the mean of the two methods, for participants included in the validation analysis. **Fig. S3**. Scatter plot with crude CO_2_e assessed by the first Meal-Q on the vertical axis and crude CO_2_e assessed by the second Meal-Q on the horizontal axis, for participants included in the reproducibility analysis. **Fig. S4**. Bland-Altman plot showing the difference in crude CO_2_e assessed by the first and second Meal-Q plotted against the mean of the two methods, for participants included in the reproducibility analysis. (DOCX 56 kb)

## Abbreviations

CO_2_e: Carbon dioxide equivalents
FFQ: Food frequency questionnaire
GHGE: Greenhouse gas emission
GWP: Global warming potential
LCA: Life cycle assessment
PAL: Physical activity level
WFR: Weighed food record
