# Supplementary material for: Diet-related greenhouse gas emissions assessed by a food frequency questionnaire and validated using 7-day weighed food records
Source: Environ Health. 2016 Feb 9;15:15. doi: 10.1186/s12940-016-0110-7 (PMC4748591; doi:10.1186/s12940-016-0110-7)
Supplement: Additional file 1: Table S1. — Data from life cycle assessment (LCA) studies expressed as carbon dioxide equivalents (CO2e) per kg of food product. Fig. S1. Scatter plot with crude CO2e assessed by Meal-Q on the vertical axis and crude CO2e assessed by WFR on the horizontal axis, for participants included in the validation analysis. Fig. S2. Bland-Altman plot showing the difference in crude CO2e assessed by Meal-Q and the WFR plotted against the mean of the two methods, for participants included in the validation analysis. Fig. S3. Scatter plot with crude CO2e assessed by the first Meal-Q on the vertical axis and crude CO2e assessed by the second Meal-Q on the horizontal axis, for participants included in the reproducibility analysis. Fig. S4. Bland-Altman plot showing the difference in crude CO2e assessed by the first and second Meal-Q plotted against the mean of the two methods, for participants included in the reproducibility analysis. (DOCX 56 kb) [file 12940_2016_110_MOESM1_ESM.docx]

Additional file 1

**Diet-related greenhouse gas emissions assessed by a food frequency questionnaire and validated using 7-day weighed food records**

Camilla Sjörs^1*^, Sara E Raposo^2,†^, Arvid Sjölander^1^, Olle Bälter^3,4^, Fredrik Hedenus^5^ and Katarina Bälter^1,6^

^1^Department of Medical Epidemiology and Biostatistics, Karolinska Institutet, SE-171 77 Stockholm, Sweden. ^2^Current address: Department of Nutrition, Harvard T.H. Chan School of Public Health, Boston, MA, 02115, USA. ^3^School of Computer Science and Communication, KTH - Royal Institute of Technology, SE-100 44 Stockholm, Sweden. ^4^Stanford Graduate School of Education, Stanford, CA, USA. ^5^Department of Energy and Environment, Chalmers University of Technology, SE-412 96 Gothenburg, Sweden. ^6^Stanford Prevention Research Center, Stanford School of Medicine, Stanford, USA.

E-mail addresses: Camilla.Sjors@ki.se; Sara.Raposo@ki.se; Arvid.Sjolander@ki.se; ob1@kth.se; Hedenus@chalmers.se; Katarina.Balter@ki.se

*Correspondence: Camilla.Sjors@ki.se

Department of Medical Epidemiology and Biostatistics, Karolinska Institutet, Nobels väg 12a, SE-171 77 Stockholm, Sweden.

**Table S1 Data from life cycle assessment (LCA) studies expressed as carbon dioxide equivalents (CO_2_e) per kg of food product**

We adjusted original LCA data to include the same system boundaries, for example added standard emissions factors from post-farm processes, including processing, packaging, distribution and retail (emissions after the retail phase, such as transports, storing and cooking, as well as from waste management were not included). LCA data were recalculated for weight change during food preparation, considering both hydration, i.e. cooking of rice, and dehydration, i.e. cooking of meat, and adjusted for unavoidable food losses (i.e. shell and bone) as well as for avoidable food losses both before and after food preparation.

| **Food products or groups** | **Kg CO_2_e / kg food product** | **Reference** |
| --- | --- | --- |
| Mutton (sheep meat), fried | 57.37 | Bryngelsson et al [1] |
| Beef, fried/boiled | 47.47 | Bryngelsson et al [1] |
| Minced meat (mix of beef and pork 70/30), fried | 37.27 | Estimated from Bryngelsson et al [1] |
| Shrimp and lobster without shell, boiled | 31.26 | Ziegler et al [2] |
| Fresh fruit, berries and vegetables, aviation | 18.48 | Röös [3] |
| Shrimp and lobster with shell, boiled | 11.88 | Ziegler et al [2] |
| Butter | 11.88 | Bryngelsson et al [1] |
| Hard/yellow cheese (incl. halloumi) | 10.95 | Bryngelsson et al [1] |
| Pork fried/boiled, ham | 10.63 | Bryngelsson et al [1] |
| Butter blends (Bregott) | 10.48 | Estimated from Bryngelsson et al [1] and Flysjö [4] |
| Soft/white cheese (incl. feta, mozzarella, cream cheese, moldy cheese) | 9.96 | Estimated from Bryngelsson et al [1] and Flysjö [4] |
| Cream | 7.90 | Estimated from Bryngelsson et al [1] and Flysjö [4] |
| Distilled beverages/ vodka/ rum | 7.59 | Estimated from Bryngelsson et al [1] |
| Salmon, fried/boiled/smoked | 7.13 | Ziegler et al [5] and Winther et al [6] |
| Game, fried/boiled | 6.70 | Cejie [7] |
| Cod, fried/boiled | 6.04 | Ziegler et al [5] and Winther et al [6] |
| Mussels, without shell | 5.68 | Ziegler et al [5] and Winther et al [6] |
| Dried fruit | 5.58 | Estimated from Bryngelsson et al [1] |
| Seafood other, fried/boiled | 5.54 | Estimated from Ziegler et al [5] and Winther et al [6] |
| Dairy, other (incl. cottage cheese) | 5.22 | Estimated from Bryngelsson et al [1] and Flysjö [4] |
| Sugar and syrup | 4.83 | Bryngelsson et al [1] |
| Foam sweets | 4.12 | Nilsson et al [8] |
| Poultry fried/boiled | 3.98 | Bryngelsson et al [1] |
| Jam | 3.79 | Röös [3] |
| Quorn | 3.73 | Röös [3] |
| Olives | 3.36 | Florén et al [9] |
| Milk chocolate | 2.91 | Nilsson et al [8] |
| Sausages fried/boiled | 2.70 | Florén et al [9] |
| Ice-cream | 2.60 | Nilsson et al [8] |
| Jelly sweets | 2.59 | Nilsson et al [8] |
| Banana | 2.58 | Bryngelsson et al [1] |
| Snacks (crisps etc) | 2.32 | Nilsson et al [8] |
| Wine / liqueur | 2.30 | Bryngelsson et al [1] |
| Vegetable oils and margarine | 2.30 | Bryngelsson et al [1] |
| Mackerel, fried | 1.83 | Ziegler et al [5] and Winther et al [6] |
| Tofu, soy sausage etc | 1.56 | Röös [3] |
| Milk, sour milk and yoghurt | 1.55 | Bryngelsson et al [1] |
| Cucumber | 1.45 | Davis et al [10] |
| Cookies and biscuits | 1.41 | Estimated from Bryngelsson et al [1] and Nilsson et al [8] |
| Herring, fried/boiled/pickled | 1.39 | Ziegler et al [5] and Winther et al [6] |
| Nuts and Seeds | 1.33 | Bryngelsson et al [1] |
| Fresh fruits and berries, imported (other than banana and citrus fruit) | 1.32 | Bryngelsson et al [1] |
| Eggs | 1.26 | Bryngelsson et al [1] |
| Cider, strong | 1.12 | Estimated from Bryngelsson et al [1] |
| Beer | 1.09 | Bryngelsson et al [1] |
| Tomato | 0.99 | Bryngelsson et al [1] |
| Dark chocolate | 0.99 | Nilsson et al [8] |
| Citrus fruit | 0.96 | Bryngelsson et al [1] |
| Juice | 0.91 | Röös [3] |
| Broccoli and vegetables not included in "root vegetables, onion" | 0.85 | Bryngelsson et al [1] |
| Rice, boiled | 0.74 | Bryngelsson et al [1] |
| Breakfast cereals | 0.71 | Bryngelsson et al [1] |
| Bread and crisp bread | 0.67 | Bryngelsson et al [1] |
| Flour, grain | 0.60 | Bryngelsson et al [1] |
| Iceberg lettuce | 0.41 | Bryngelsson et al [1] |
| Soy drink, oat drink, coconut milk | 0.30 | Bryngelsson et al [1] |
| Fresh fruit and berries, domestic | 0.30 | Bryngelsson et al [1] |
| Legumes, soaked and boiled | 0.29 | Bryngelsson et al [1] |
| Potatoes, boiled/baked/fried/french fries | 0.29 | Bryngelsson et al [1] |
| Pasta, couscous, bulgur, quinoa, boiled | 0.27 | Bryngelsson et al [1] |
| Root vegetables, onion | 0.24 | Bryngelsson et al [1] |
| Coffee | 0.18 | Nilsson [11] |
| Soft drinks, fruit syrup | 0.17 | Nilsson et al [8] |
| Tea | 0.04 | Estimated from Nilsson [11] and Scarborough et al [12] |
| Unknown | 0 |  |

**References Table S1**

1. Bryngelsson D, Wirsenius S, Hedenus F, Sonesson U. How can the EU climate targets be met? A combined analysis of technological and demand-side changes in food and agriculture. Food Policy (2016), http://dx.doi.org/10.1016/j.foodpol.2015.12.012

2. Ziegler F, Valentinsson D. Environmental life cycle assessment of Norway lobster (Nephrops norvegicus) caught along the Swedish west coast by creels and conventional trawls—LCA methodology with case study. Int J Life Cycle Assess. 2008;13(6):487-97. doi:10.1007/s11367-008-0024-x.

3. Röös E. Mat-klimat-listan Version 1.0 (Food-climate-list Version 1.0). In Swedish. Swedish University of Agricultural Sciences (SLU). 2012. Report 040. ISSN 1654-9406. [Internet]. Available: http://pub.epsilon.slu.se/8710/1/roos_e_120413.pdf Archived at: http://www.webcitation.org/6XsldtvS1 Accessed August 19 2015

4. Flysjö AM. Greenhouse gas emissions in milk and dairy product chains: Improving the carbon footprint of dairy products. PhD thesis. Aarhus University, Denmark. 2012.

5. Ziegler F, Winther U, Hognes ES, Emanuelsson A, Sund V, Ellingsen H. The carbon footprint of Norwegian seafood products on the global seafood market. Journal of Industrial Ecology. 2013;17(1):103-16.

6. Winther U, Ziegler F, Hognes ES, Emanuelsson A, Sund V, Ellingsen H. Carbon footprint and energy use of Norwegian seafood products. SINTEF Fisheries and Aquaculture, Report SFH80 A. 2009;96068.

7. Cejie J. Klimatpåverkan från vilt kött (Climate impact from game meat). [Internet]. In Swedish. 2008. http://ekotank.blogspot.se/2008/11/klimatpverkan-frn-vilt-ktt.html Archived at: http://www.webcitation.org/6RfsoOEpH Accessed August 19 2015

8. Nilsson K, Sund V, Florén B. The environmental impact of the consumption of sweets, crisps and soft drinks. Copenhagen: Nordic Council of Ministers 2011. TemaNord 2011:509.

9. Florén B, von Bah B, Davis J, Flysjö A, Högberg J, Lorentzon K et al. Global warming potential for 100 ICA private label food products. 2007. Final report UP-07-14423.

10. Davis J, Wallman M, Sund V, Emanuelsson A, Cederberg C, Sonesson U. Emissions of greenhouse gases from production of horticultural products. Analysis of 17 products cultivated in Sweden. SIK - The Swedish Institute for Food and Biotechnology, Gothenburg, Sweden. 2011. SR 828. ISBN 978-91-7290-301-2

11. Nilsson K. Klimatpåverkan från bryggkaffe och snabbkaffe (Climate impact of filter coffee and instant coffee). In Swedish. Report UPX00221. SIK - The Swedish Institute for Food and Biotechnology, Gothenburg, Sweden. 2010.

12. Scarborough P, Appleby PN, Mizdrak A, Briggs ADM, Travis RC, Bradbury KE et al. Dietary greenhouse gas emissions of meat-eaters, fish-eaters, vegetarians and vegans in the UK. Climatic Change. 2014. doi:10.1007/s10584-014-1169-1.

|   **Fig. S1** Scatter plot with crude CO_2_e assessed by Meal-Q on the vertical axis and crude CO_2_e assessed by WFR on the horizontal axis, for participants included in the validation analysis (*n*=166). The outlier to the right is a person on a low carbohydrate high fat diet.  CO_2_e, carbon dioxide equivalents. WFR, weighed food record |
| --- |

|   **Fig. S2** Bland-Altman plot showing the difference in crude CO_2_e assessed by Meal-Q and the WFR plotted against the mean of the two methods, for participants included in the validation analysis (*n*=166). Each data point represents one subject. The grey background show the 95% limits of agreement. CO_2_e, carbon dioxide equivalents. WFR, weighed food record |
| --- |

|   **Fig. S3** Scatter plot with crude CO_2_e assessed by the first Meal-Q on the vertical axis and crude CO_2_e assessed by the second Meal-Q on the horizontal axis, for participants included in the reproducibility analysis (*n*=87). CO_2_e, carbon dioxide equivalents |
| --- |

|   **Fig. S4** Bland-Altman plot showing the difference in crude CO_2_e assessed by the first and second Meal-Q plotted against the mean of the two methods, for participants included in the reproducibility analysis (*n*=87). Each data point represents one subject. The grey background show the 95% limits of agreement. CO_2_e, carbon dioxide equivalents |
| --- |
